# The use of video consultations to support orthopedic patients' treatment at the interface of a clinic and general practitioners

**DOI:** 10.1186/s12891-022-05909-2

**Published:** 2022-11-08

**Authors:** Estel K, Richter L, Weber G, Fellmer F, Märdian S, Willy C, Back DA

**Affiliations:** 1 Department for Traumatology and Orthopedics, Bundeswehr Hospital Berlin, Scharnhorststrasse 13, 10115 Berlin, Germany; 2 Medical Faculty, Charité - Universitätsmedizin Berlin, Corporate Member of Freie Universität Berlin and Humboldt-Universität zu Berlin, Charitéplatz 1, 10117 Berlin, Germany; 3 Center for Musculoskeletal Surgery, Charité - Universitätsmedizin Berlin, Corporate Member of Freie Universität Berlin and Humboldt-Universität zu Berlin, Augustenburger Platz 1, 13353 Berlin, Germany; 4 Dieter Scheffner Center for Medical Education and Educational Research, Charité - Universitätsmedizin Berlin, Corporate Member of Freie Universität Berlin and Humboldt-Universität zu Berlin, Charitéplatz 1, 10117 Berlin, Germany

**Keywords:** Video consultation, Orthopedics, Support, Clinic, General practitioners

## Abstract

**Introduction:**

Video consultations have proven to be a powerful support tool for patient–doctor interactions in general, not only during the COVID-19 pandemic. This study analyzed the feasibility and usefulness of orthopedic telemedical consultations (OTCs) for orthopedic patients at the interface of a clinic and general practitioners.

**Methods:**

The study was carried out at an orthopedic department of a German hospital between April 2020 and October 2020. After written informed consent was obtained, general practitioners (GPs) of a large adjunct health region could present their patients with orthopedic pathologies to specialists at the hospital via OTCs instead of the usual live consultation (LC). The patients, specialists and GPs were evaluated for their OTC experience and attitude (5-point Likert-scale and open questions, 19 to 27 items).

**Results:**

A total of 89 video consultations took place with 76 patients, 16 GPs and six specialists. The average distance between the GPs/patients and the hospital was 141.9 km. The OTCs were rated as pleasant, and the experience was rated as very satisfying (average Likert-Scale rating, with 5 as strong agreement: specialists = 4.8; GPs = 4.9; patients = 4.7). Following the OTC, a LC was not necessary in 76.4% of cases. Patients with a necessary LC after an OTC showed significantly lower satisfaction with the OTC (*p* = 0.005). Time savings, the elimination of travel and quick contact with orthopedic consultants were positively highlighted by the participants. A total of 123 recommendations for further treatment were given, such as the initiation of physiotherapy/medication and the use of imaging diagnostics. Different technical and organizational challenges could be identified and addressed.

**Discussion:**

The vast majority of the participants stated they had a very positive impression. In particular, the potential savings in travel and time as well as straightforward contact with specialists were rated positively. However, limitations in the assessment of initial presentations of complex medical conditions were also highlighted. Further studies on OTCs with a consultative health professional may show other fields of use for this mode of interdisciplinary remote communication.

**Supplementary Information:**

The online version contains supplementary material available at 10.1186/s12891-022-05909-2.

## Introduction

Telemedicine is a powerful tool in digitalization in the health sector. It offers the advantage of transferring the medical expertise of specialists over long distances with a reduction in both travel and waiting times for patients [1, 2]. As a part of telemedicine, the goal of video consultations is to enable medical contact between health professionals and patients—just as physical presentations in live consultations (LCs) [1]. In recent years, sufficient technology and legal foundations have been established, so video consultations are already being used in many countries' daily practices [3]. Furthermore, the current SARS-CoV-2 (COVID-19) pandemic has increased the need to offer noncontact treatment options and disseminate their acceptance among patients and physicians [4].

Recent studies have already demonstrated a high acceptance among patients for telemedical doctor visits [3]. In addition, medical staff consider the results of orthopedic telemedical consultations (OTCs) to be equivalent to those of LCs [5]. However, some studies have also stated that technical problems or a lack of knowledge regarding data exchange represent possible reservations of medical staff [6, 7].

An additional benefit of OTCs may be seen in the enhancement of collaborations between specialists (e.g., at the clinic) and general practitioners (GPs) in the aftercare of patients [8]. Since patients are often seen first by their GPs and are only transferred to a specialist in cases of more complex pathologies or to seek other advice, digital means may be used to support these existing structures. For example, study data have shown that the number of LCs with specialists could be reduced when a primary care physician preselects orthopedic consultation requests and provides an OTC between a patient and a specialist [2].

Different articles have been published reporting the clinical use of OTCs and the positive attitudes and experiences of orthopedic surgeons and patients [9, 10]. However, data describing the benefits of a consultative presentation of patients to orthopedic specialists by various primary care physicians of a health region through OTCs and with subsequent treatment planning via OTCs are still lacking [2].

This work aimed to extend established OTCs in an orthopedic hospital department to the care of patients in general practice in the adjunct health region of the hospital. In particular, the feasibility, quality, benefits and acceptance of OTCs at the interface between the clinic and general outpatient practice was analyzed.

## Materials and methods

### Study design and setting

OTCs were introduced as a pilot project in the Bundeswehr Medical Service and the Bundeswehr Cyber Innovation Hub in Germany at the Department of Traumatology and Orthopedics of the Military Academic Hospital Berlin. Military physicians at regional medical facilities (comparable to civilian general practitioners, subsequently referred to as "GPs") were equipped with the necessary technology to present patients with orthopedic pathologies to a hospital specialist or an orthopedic specialist affiliated with the hospital. These orthopedic specialists were the main point of contact for many soldiers in eastern Germany, so sometimes long distances had to be travelled for a standard live consultation (LC). In this project, the patients' GPs could choose between a digital consultation or the “traditional” LC. Appointments were set by telephone, email or via the program used by the provider. Patients gave their informed consent with the option to withdraw from the study at any time without giving reasons and without consequences for their treatment before inclusion. The acquired data were pseudonymized. Data from the patients concerning their medical history, examination, diagnosis, and recommended procedure were documented using the standardized regular report forms of the hospital information system (HIS) during the consultation—analog to LCs. After the online appointment, where GP together with their patients on the one side and the specialist orthopedic surgeons (12 between patients and specialists without GPs), the GPs and specialists were asked to assess their examination experience using an evaluation sheet and the patients were asked to assess their consultation experience. The study period was April 2020 to October 2020, with prior approval of the responsible ethics committee of the Berlin Medical Association ( Ärztekammer Berlin, No: Eth-12/19).

### Technical procedure

Following the requirements of the national jurisdiction, only real-time videoconferencing was conducted with the participants. After a certified provider was selected (Deutsche Arzt AG, Essen, Germany), the provider established secure broadband connections between the patients and physicians. Before each OTC, a link and a password were sent via email or SMS to the GPs or patients. Herewith, they obtained access to a protected virtual waiting area. Once the physician was online, the OTC was started in a separate virtual room. The system used a browser-based approach; hence, the connection was not restricted to a specific device (cell phone, tablet, etc.), operating system or manufacturer.

#### Participation criteria

All study participants were soldiers of the German Federal Armed Forces beyond 18 years of age and were versed in internet-based communication. Another prerequisite was a regular course of medical treatment. Exclusion criteria were, apart from the personal decision against participation, any circumstances in which—in the opinion of the treating doctors—continued involvement in the study would not have been in a patient's best interest.

### Structure of the physicians' questionnaires

The questionnaire for the specialists contained 27 questions (closed 5-point Likert-scale questions with 1- Fully agree until 5 strongly disagree, half-open and open questions), which were divided into the following blocks:

Demographic data and preferences for digital services (five questions)

General questions about the experience of the OTC: punctuality, technical problems with contact, atmosphere (four questions),

Details on findings from the OTC as well as their recommended procedures (six questions)

Specific questions on the experience of the OTC: assessment of sound/image quality, examination procedure, satisfaction with the OTC, advantages and disadvantages, and potential for optimization (twelve questions).

The questionnaire for the GPs contained 26 questions (closed 5-point Likert-scale questions, half-open and open questions), which were divided into the following blocks:

Demographic data and preferences for digital services (nine questions)

General questions about the experience of the OTC: punctuality, technical problems with contact, atmosphere (four questions)

Specific questions on the experience of the OTC: assessment of sound/image quality, examination procedure, satisfaction with the OTC, advantages and disadvantages, and potential for optimization (thirteen questions).

The questionnaire for patients contained 19 questions (closed 5-point Likert-scaled questions, half-open and open questions), which were divided into the following blocks:

Demographic data (two questions)

General questions about the experience of the OTC: punctuality, technical problems with contact, atmosphere (three questions)

Specific questions about the experience of the OTC: sound/image quality, examination procedure, improvement options (eleven questions)

Questions comparing the OTC to a standard LC: distance and means of transportation (three questions)

Participants who did not wish to participate were planned to be verbally asked about their reasons for declining.

### Data analysis

Quantitative analysis: Data of this evaluation study were recorded with Excel (Version 2016, Microsoft Inc., Redmond, WA, USA). For the questionnaire, quantitative data analysis with evaluation according to descriptive statistical methods was performed. Data distribution analysis was performed by Schiefe und Kurtosis and the Kolmogorov–Smirnov and Shapiro–Wilk test measures. Statistical significance was then determined with the Mann–Whitney U Test (SPSS, Version 27.0.1, IBM, New York, United States).

Within the statistical evaluation, one specific aspect of interest was the detection of possible factors influencing the measured satisfaction of the participating patients with the OTC experience. The data on satisfaction with the OTC were therefore analyzed for this purpose in relation to travel savings through the OTC, the need for an outpatient clinic visit subsequent to the O-VS, and the presence of problems.

Qualitative analysis: Furthermore, free text answers were frist summarized (summary base form) and analysed, and then examined by two independent authors for recurring statements with systematic qualitative content analysis, according to Mayring [11]. Subsequently, both authors independently interpreted, concluded, and summarized the findings.

## Results

A total of 89 OTCs were included in the study (77 first presentations, nine second presentations, one patient with a third and fourth presentation, one between a specialist and GP).

The majority of the OTCs occurred with one patient, one specialist, and one GP present at the same time (*n* = 77). Twelve OTCs were performed between a patient and a specialist alone, but with the indication and consent of the responsible GP.

**Fig. 1:**
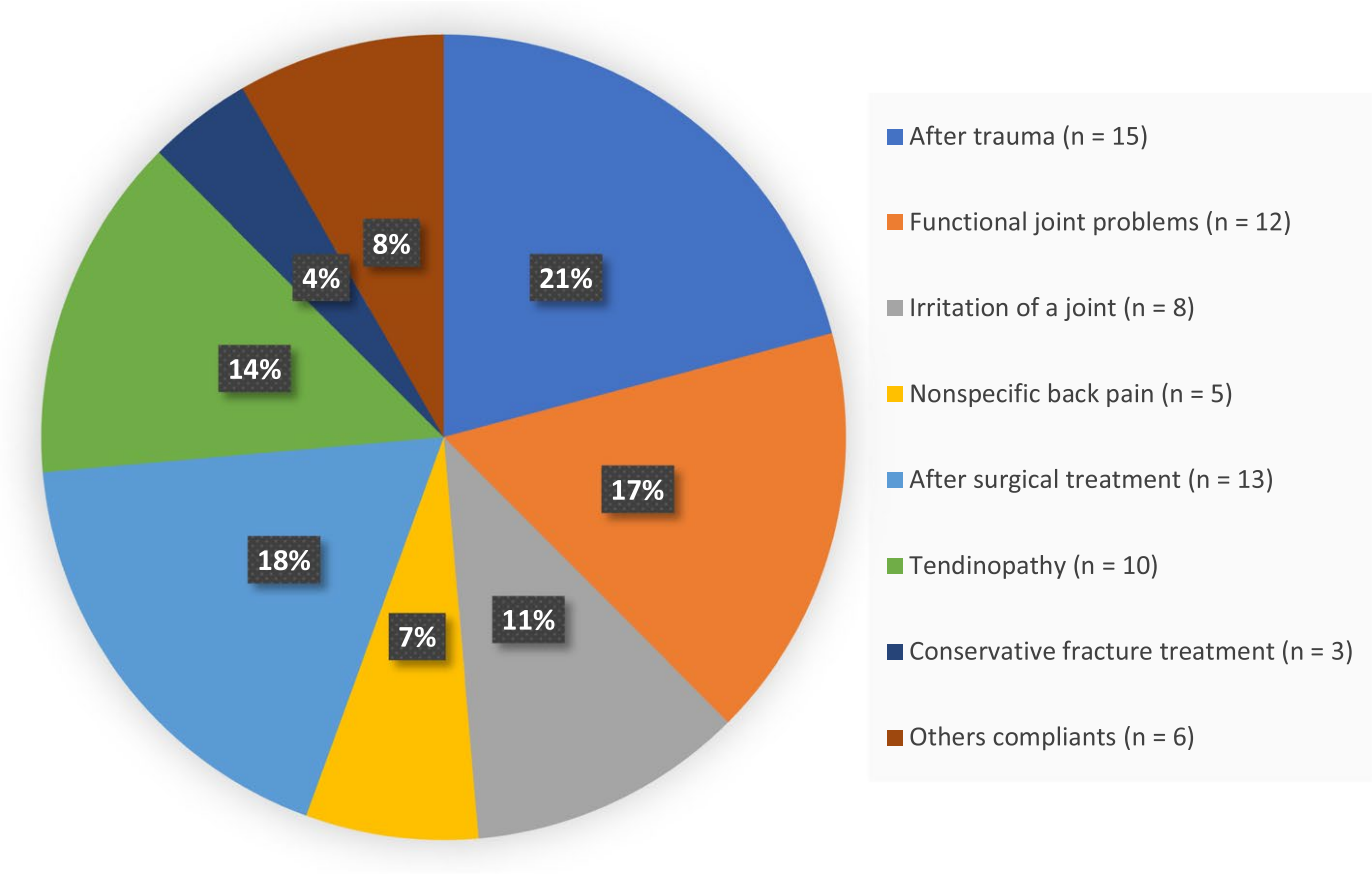
Classification of patient cases (*n* = 72) into groups according to their pathologies

Most OTCs took place in the regional medical facility (*n* = 77). Only a small number were completed from patients' homes (*n* = 9) or other locations (*n* = 3). The different orthopedic pathologies are categorized to major clinical pictures and summarized in Fig. 1. No patient who was offered the opportunity refused to participate.

### Evaluation of specialists` perceptions

A total of 6 orthopedic specialists (m:f 4:2) participated. Their average age was 38.8 (± 1.6) years (Table 1).

**Table 1:** Presentation of the demographic data of all participants (*n* = 98)

Group of population	Average age	Amount of participants	Male	Female
*Orthopedic specialists*	*38.8*	*6*	*4*	*2*
*GPs*	*34.1*	*16*	*5*	*11*
*Patients*	*37.2*	*76*	*64*	*12*

**Fig. 2:**
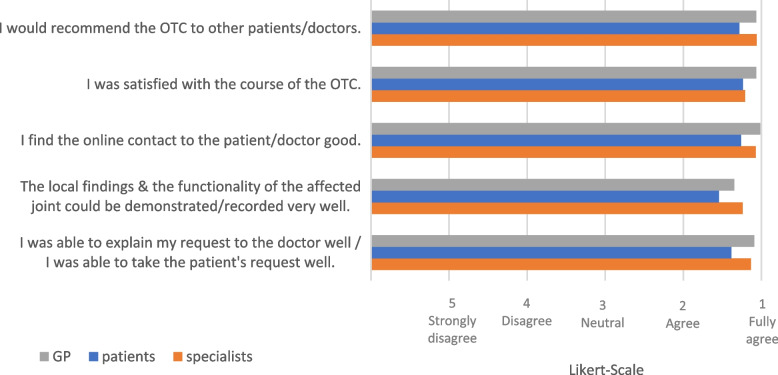
Evaluation of the experience of the performed OTCs (*n* = 89) by GPs, patients and specialists (5-point Likert scale)

All participating specialists described the atmosphere during the OTC as "very pleasant" (*n* = 89). The detailed evaluation results of the specialists' OTC experiences are shown in Fig. 2. In free text answers (not included in Fig. 2), time savings (*n* = 3) and an acceleration of further procedures for patients (*n* = 2) were indicated by the six specialists as the most important benefits for their own practice.

In 64 cases, the specialist did not experience any problems with the OTC. Registered general technical issues or problems with the OTC are described in more detail below.

### Evaluation of the GPs’ perceptions

Sixteen GPs (m:f 5:11) with an average age of 34.1 (± 6) years participated in the assessment (Table 1). Concerning the OTCs, the GPs evaluated almost all consultations as "very pleasant" (*n* = 72). Figure 2 provides the detailed assessment results of this group. This occupational group emphasized the option for short notice contacts and further inquiries (*n* = 3), fast decision-making (*n* = 4), increased training (*n* = 3) and no lack of information flow (*n* = 2). In 37 OTCs of this cohort, no problems occurred. A summary of the issues is shown in Fig. 4.

### Evaluation of patients' perceptions

The mean age of the 76 participating patients (m:f 64:12) was 37.2 (± 9.7) years (Table 1). The OTCs were rated concerning the atmosphere as either “immediately pleasant” (*n* = 53) or “first unfamiliar, but pleasant in the following” (*n* = 35). The remaining evaluation results of the patients' OTC experiences are shown in Fig. 2. The patients cited time savings (*n* = 13), the elimination of travel distance (*n* = 5), reduced travel costs (*n* = 3), and reduced waiting time (*n* = 2) as the primary benefits. At the time of the OTC, the average distance between the patients and treatment location was 141.9 km [1 to 650 km]. The patients reported no problems during the OTC in 60 cases.

### OTC-based treatment recommendations for patients

Concerning assessment of the benefits of OTCs, the specialists (*n* = 85) indicated, in 95% of the cases, that it was the correct type of contact. In 4 OTCs, the specialists were unsure whether an OTC was indicated. In 21 OTCs, the patients also had to present to the outpatient clinic. These patients were significantly less satisfied with the OTC than the patients without outpatient clinic appointments (*p* = 0.005).

**Fig. 3:**
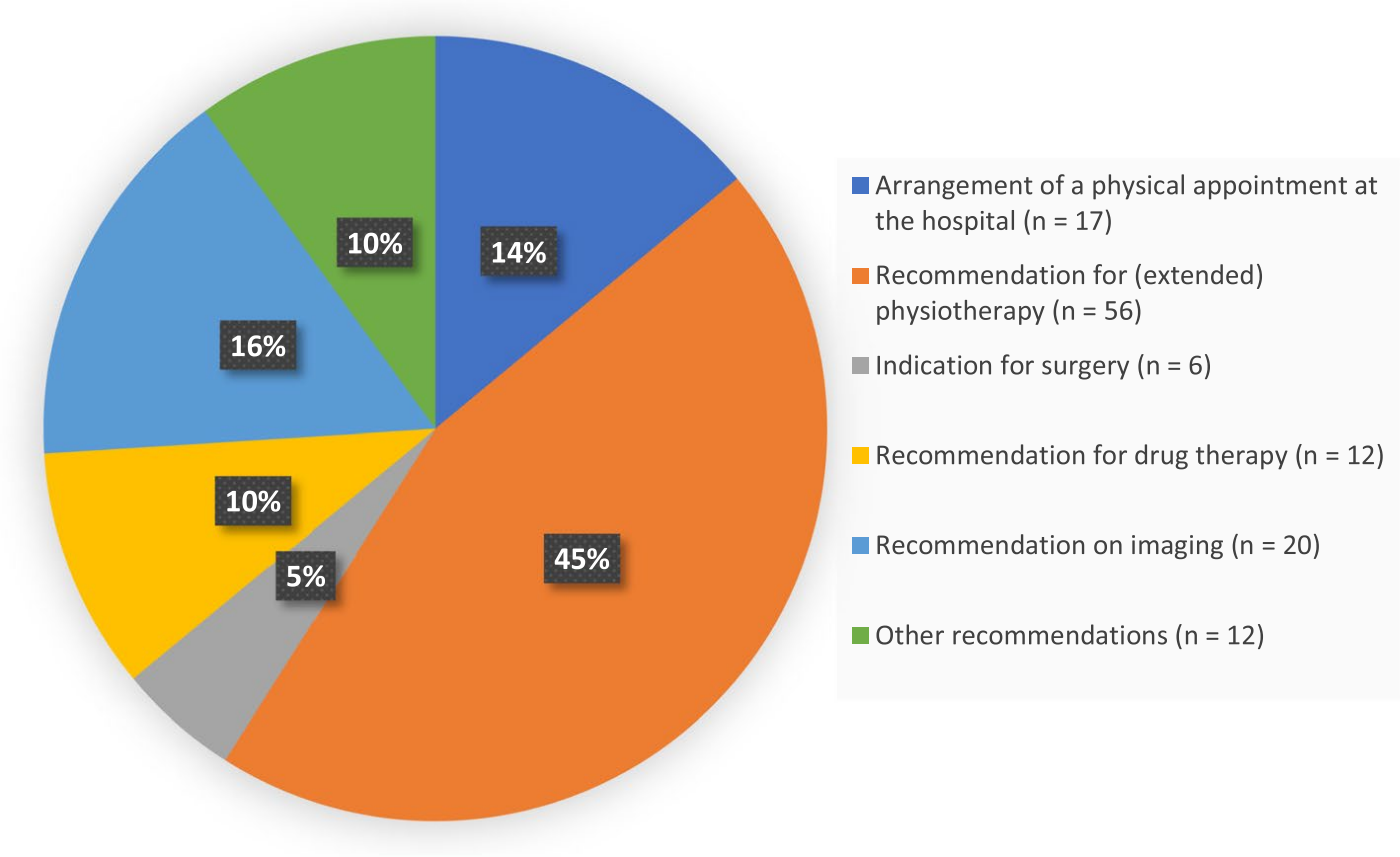
Presentation of the recommendations that the specialists reported for the patients in all OTCs (*n* = 89) according to 123 statements of the specialists

The procedural recommendations that emerged from the consultations are shown in Fig. 3. For a total of twelve patients, there were “other" recommendations: orthopedic insoles (*n* = 7) and bandages (*n* = 2), as well as presentation to another specialist discipline (*n* = 1), referral to a civilian surgeon (*n* = 1) or workplace adjustment (*n* = 1).

### OTC technical difficulties

**Fig. 4:**
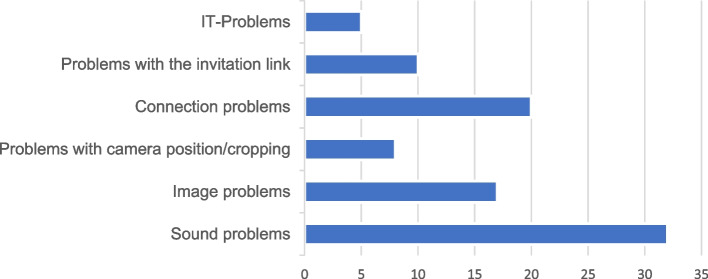
Accurate representation of technology problems (*n* = 92) considering all evaluations (*n* = 256) (IT problems = information technology problems, here: functional problems with the OTC provider software used)

Figure 4 provides an overview of the problems (*n* = 96) during the OTCs, which were primarily of a technical nature (*n* = 92).

Overall, the specialists and GPs saw a need for improvement in the organizational structure of the OTC (specialists: *n* = 8; GPs: *n* = 3), improved sound and image quality (specialists: *n* = 8; GPs: *n* = 3), a more stable internet connection (specialists: *n* = 7; GPs: *n* = 10), improved hardware with additional keyboards for the tablets and tablet holders with a better operability (specialists: *n* = 0; GPs: *n* = 5), better possibilities of transmitting radiological images, not only their findings (specialists: *n* = 0; GPs: *n* = 3) and more OTC pretraining for GPs (specialists: *n* = 3; GPs: *n* = 1).

## Discussion

In the current transformation of digital health care and the COVID-19 pandemic, digital options for adequate patient care over distances have become increasingly important [3, 9, 12], including in specialties with predominantly hands-on examinations, such as orthopedics [10, 13, 14]. In this context, only a few studies have described patients' digital presentations by their GPs to specialists [8], especially in orthopedics [2].

The present study examined the benefits of OTCs among orthopedic specialists, GPs and patients, covering technical feasibility, assessments of usefulness, quality and possible obstacles and challenges. A unique feature of this study was the interaction and corporate treatment of patients by GPs **and** orthopedic specialists by digital OTCs, in most cases **with** the patients´ live participation.

The study demonstrated a high level of satisfaction among the participating groups with the consultative OTC. Additionally, close contact between the general practitioners and specialists was particularly valued. In addition, time savings by the elimination of travel and quick contact with orthopedic consultants were positive aspects of the OTC. In 64% of cases, recommendations could be made without the need for an LC.

The results demonstrate that the majority of the participants evaluated their OTC experience as positive. In particular, the atmosphere of the OTC was perceived as "very pleasant" or "…pleasant in the following". The finding that more patients than physicians rated the experience as “…pleasant in the following" may be explained by a natural reaction when they were exposed to an initially unknown physician "on the screen" with a previously rather unfamiliar digital communication method. Another possible explanation might be the concern of the impairment of the doctor–patient relationship due to the lack of in-person contact that has already been described in previous studies, which is why patients approach the OTC experience somewhat cautiously [15].

The experience of the OTC was also mainly rated as very satisfactory by all participant groups, which is in concordance with results published elsewhere [16]. However, although the group of patients rated the OTC experience very positively, slight deviations from the physicians were observed. In particular, the presentation of local findings and functionality were evaluated slightly less favorably by the patient group than the two groups of physicians. It has already been published that the physical examination of patients with musculoskeletal disorders via telemedicine is limited [17, 18]. However, with special tools and an adaptation of the examination techniques to the digital setting, quite a large number of functional joint assessments could also be performed sufficiently via OTCs [19]. Nevertheless, many published digital examination solutions lack evidence for their efficacy, which must be elaborated in future studies [17, 18, 20].

Particularly noteworthy is the crucial factor that an OTC could reduce travel distances for patients, which averaged 141 km in this study and could thus result in relevant time savings. Previous studies demonstrated that this aspect [1], combined with the elimination of long waiting times [2], is positively received by patients. In addition, the use of an OTC can be cost-minimizing by saving travel costs as well as preventing the absence of patients from their workplaces [21]. Furthermore, it is known that patient compliance with follow-up routines is higher when travel distances are low [22]. Hence, it might be assumed that OTC follow-up appointments are more likely to be attended [22].

LC follow-ups were required after approximately one-fifth of the performed OTCs, consistent with the results of previous studies [2]. In addition, most of the patients with LCs underwent a surgical procedure. Therefore, they had to present in person to review the surgical indication and/or preop preparation (legal consent, anesthesia, etc.). In summary, the subsequent LC was beneficial for these patient and helped to initiate a timely and satisfactory treatment course.

The GPs’ questions about further imaging, physiotherapy or drug therapy were very often answered satisfactorily in the context of the OTC. They can therefore be considered relevant reasons for the presentation of orthopedic patients for OTCs. To the best of our knowledge, there are no data concerning this issue that are available to date.

In addition to the various positive aspects for the participants, our data show disruptive influences (mainly technical issues) comparable to results published previously [1, 23]. While no problems occurred in approximately two-thirds of the OTCs, one-third of the OTCs had difficulties with the image, sound, connection, camera settings, hardware or receipt of the invitation link, which should be avoided in the future to improve users´ acceptance [24]. Additionally, further potential limitations of telemedical consultations should be noted, since especially legal, safety or also data security issues are still partially or completely unresolved in many countries. Therefore, save technical and clinical solutions should be developed, taking these aspects into recommendation in particular [25].

With regard to the collaboration between GPs and specialists via OTCs, the results presented here show that the digital form of consultative patient presentation might positively impact the relationship between the different groups. The clear advantages were short-term contact and improved information flow through direct communication, which is not the case with the conventional process. In addition, there was the possibility of questions raised by the GPs being directly answering, which increases understanding and has a continuing educational role.

This form of consultation represents a unique opportunity for interdisciplinary collaborations that would be desirable for intercollegiate confidence building and for the benefit of the patients.

However, the here presented form of remote communication with two different specialized doctors and a patient is rather not likely to be a standard procedure, but reserved for special indications. Reasons might be found in various logistical problems with the coordination of the schedule of two busy physicians being available at the same time in different places. Also financial aspects with more or less double costs for a physician consultation could be relevant in both the military and civilian sectors.

The study also has some limitations. For example, the young average age of the participants and the potentially associated general affinity for digitization topics might have led to positive selection biases in the survey, since elderly patients might be less well versed in internet-delivered communication. More participants with a broader age spectrum and a balanced sex distribution should be included in the future. In addition, the number of selected participants, both patients and physicians, was not comprehensive enough to make general statements about the suitability of OTCs with this interdisciplinary approach.

In the future, it may be recommendable to extend the a specialist consultation OTC to other medical areas, where the focus is on taking the patient's medical history and/or inspection. For the field of orthopedics, more usecases and scientific evidence by randomized controlled trials still have to be published on useful substitutional examination techniqeus for e.g. testing of joint functions, such as shoulder or knee instabilities.

Useful ammendments to OTCs and an enrichment of digital medicine could also be the use of digital health tools, such as data transmission from the patient to its doctors by wearables or mobile apps or also the us of virtual, augmented and orther forms of mixed reality. This can lead to an expansion of the available data and the medical network with improved collaboration between GPs and specialists fort he sake of their patients.

## Conclusion

Based on the results of this study with high satisfaction among the participating groups, it seems beneficial to perform consultative OTCs involving specialists, general practitioners and patients for orthopedic complaints. In addition to the already published positive aspects of OTCs, the presented data emphasized the close contact between GPs and specialists. Moreover, serving as digital support rather than as a replacement for live consultations, OTCs can be used to accelerate solutions for further diagnostics or treatment plans for more straightforward orthopedic case management. However, technical issues for practicable application in the daily work of physicians must be addressed, and further research on the use of this promising digital form of doctor–patient interactions should be performed in the future.

## Supplementary Information

**Additional file 1. **Questionnaire of the orthopedic specialists.

**Additional file 2. **Questionnaire of the general practitioners.

**Additional file 3. **Questionnaire of the patients.

## Data Availability

The datasets used and/or analyzed during the current study are available from the corresponding author upon reasonable request.
