# Supplementary material for: The use of video consultations to support orthopedic patients' treatment at the interface of a clinic and general practitioners
Source: BMC Musculoskelet Disord. 2022 Nov 8;23:968. doi: 10.1186/s12891-022-05909-2 (PMC9640897; doi:10.1186/s12891-022-05909-2)
Supplement: Supplementary file 3 — Additional file 3. Questionnaire of the patients. [file 12891_2022_5909_MOESM3_ESM.docx]

| **1.** | **Demographic data** |
| --- | --- |
|  | Age? _____ years  Gender ⬜ female ⬜ male |

| **3.** | **Use of the OTC** |
| --- | --- |
|  | How do you rate the contact with the counterpart as well as the atmosphere compared to a personal contact?  ⬜ Very pleasant  ⬜ At first somewhat unfamiliar, but pleasant in the course of time  ⬜ Distant and permanently alienating  ⬜ For me, the contact was unpleasant and impersonal  What were the problems with the OTC?  ⬜ Technical problems  ⬜ Finding/keeping appointments  ⬜ Problems explaining my concerns/complaints.  ⬜ Other (please specify): ____________________  ⬜ There were no problems |

| **4.** | **Specific Questions** | Fully agree | Agree | Neutral | | Disagree | | Strongly disagree |
| --- | --- | --- | --- | --- | --- | --- | --- | --- |
|  | I was able to explain my request tot he specialist well. | ⬜ | ⬜ | ⬜ | | ⬜ | | ⬜ |
|  | The local findings of the affected joint could be demonstrated very well. | ⬜ | ⬜ | ⬜ | | ⬜ | | ⬜ |
|  | The functionality of the affected joint could be demonstrated very well. | ⬜ | ⬜ | ⬜ | | ⬜ | | ⬜ |
|  | I find the online contact to the specialist good. | ⬜ | ⬜ | ⬜ | | ⬜ | | ⬜ |
|  | I was satisfied with the process of the online video consultation. | ⬜ | ⬜ | ⬜ | | ⬜ | | ⬜ |
|  |  |  |  |  | |  | |  |
|  | I would recommend the OTC to other physicians | ⬜ | ⬜ | ⬜ | | ⬜ | | ⬜ |
|  |  |  |  |  | |  | |  |
|  | What can be improved for the implementation of an OTC? (free response)  _____________________________________________________________________________  Where do you see differences (advantages/disadvantages) between online vs. "live" consultation? (free response)  _____________________________________________________________________________________________________________ | | | | | | | |
|  |  |  |  |  |  | |  | |
